# A Cocktail-Based Formula for the Design of Nanosized Cosmeceuticals as Skincare and Anti-Age Products

**DOI:** 10.3390/nano13172485

**Published:** 2023-09-04

**Authors:** Ines Castangia, Federica Fulgheri, Matteo Perra, Gianluigi Bacchetta, Laura Fancello, Francesco Corrias, Iris Usach, Josè Esteban Peris, Maria Letizia Manca, Maria Manconi

**Affiliations:** 1 Department of Life and Environmental Sciences, University of Cagliari, University Campus, S.P. Monserrato-Sestu Km 0.700, 09042 Monserrato, Cagliari, Italy; ines.castangia@unica.it (I.C.); federica.fulgheri92@unica.it (F.F.); bacchet@unica.it (G.B.); laurafancello2@gmail.com (L.F.); francesco.corrias@unica.it (F.C.); manconi@unica.it (M.M.); 2 Biomedical and Tissue Engineering Laboratory, Fundación de Investigación Hospital General Universitario, 46022 Valencia, Spain; perra_matteo@gva.es; 3 Department of Pharmacy and Pharmaceutical Technology and Parasitology, University of Valencia, Burjassot, 46100 Valencia, Spain; iris.usach@uv.es (I.U.); jose.e.peris@uv.es (J.E.P.)

**Keywords:** grape pomace, nanoemulsions, keratinocytes, raw-264-7, natural antioxidant, cosmetic antiaging

## Abstract

Nasco and Bovale grape pomace extracts, alone or in association, were loaded in nanoemulsions tailored for cosmetic application, using Kolliphor^®^RH40 (kolliphor) as the synthetic surfactant, Olivem^®^1000 (olivem) as the natural one, and lecithin as the cosurfactant. Pink transparent or milky dispersions, as a function of the used extract and surfactant, were obtained to be used as cosmeceutical serum or milk. The sizes of the nanoemulsion droplets were small (≈77 nm with kolliphor and ≈141 nm with olivem), homogenously dispersed (~0.24 with kolliphor and ~0.16 with olivem), highly negatively charged (≈−43 mV irrespective of the used surfactant) and their stability either on storage or under stressing conditions was affected by the used extract and surfactant. Formulations protected the extracts from the degradation caused by UV exposition, were biocompatible against keratinocytes, protected them against oxidative damages induced using hydrogen peroxide and inhibited the release of nitrite induced in macrophages using the lipopolysaccharide inflammatory stimulus. The overall results underlined the key role played by the composition of the formula to achieve a suitable cosmeceutical for skin care but even for the prevention of premature aging and chronic damages caused by the stressing conditions.

## 1. Introduction

The natural process of decline is well known as aging, which occurs in cells, organs or whole organisms as time goes by, and it cannot be avoided. Intrinsic factors (genotype, endocrine metabolism and hormone levels) and especially extrinsic causes (unhealthy diet, ultraviolet (UV) radiation and air pollution) affect skin aging, leading to premature effects [1,2,3]. The extrinsic factors, especially nutrition, tobacco, UV radiations or atmospheric pollution, substantially increase free radical accumulation in the skin, leading to inflammation mediated by reactive oxygen species (ROS), DNA damage, lipid peroxidation and protein crosslinking [4]. These alterations firstly address visible skin aging involving weakening of both the epidermis and dermis, reduction of elastic fibers and decrease in both collagen synthesis and the number of fibroblasts, while chronically, they improve the risk of cutaneous and systemic diseases, including tumors and, especially, skin cancer [2]. Intrinsic aging depends on the equilibrium between ROS production and the tissue capability of neutralizing them using an endogenous antioxidant system, which is less effective over time and could be saturated when these species are overproduced, causing a prepathological condition called oxidative stress [4,5]. The high accumulation of ROS could be controlled, reducing the exposure to external factors and using useful daily skincare products, which are crucial not only to improve the exterior aspect of the skin but substantially to avoid dermatological stresses and disorders [6,7]. These products can contain natural or synthetic functional molecules; the first have been used for centuries for skin care purposes, especially in Ayurveda, one of the most ancient medicines and also involving skills and practices devoted to the management of aging, health and beauty of the skin [8]. In the last decades, the use of plant-derived molecules or extracts as key components of complementary medicine or cosmetic treatments has acquired a renewed interest in developed countries due to their safety and beneficial properties. Actually, the most promising are phytocomplexes or antioxidant molecules; the first are mixtures of active and inactive molecules that act synergistically to achieve a biological effect [9].

In particular, phytocomplexes have attracted the attention of both the scientific community and industries, as they are also contained in high amounts in the so-called agri-food byproducts, obtained upon the transformation of either fruits or plants into a ready-to-use food or beverage. From these plant- and fruit-derived byproducts, it is possible to obtain extracts rich in phytocomplexes, even though the yield of recovery is strictly dependent not only on the type of residue processed but also on the type of extraction chosen [10,11]. Among the different fruits, grapes are the one cultivated the most in the Mediterranean area to produce wine, which, in turn, is associated with a huge amount of waste, called pomace, that, if not effectively managed, may represent a big environmental problem [12,13,14]. In this scenario, the use of grape pomace may represent an important strategy for the transformation of a residue into a valuable and marketable product, with beneficial properties for human health.

Antioxidants, naturally occurring in fruits, plants and other natural sources, are chemicals capable of inhibiting the oxidation of reactive molecules and inactivating free radicals [15,16]. Unfortunately, these molecules are highly unstable and have low bioavailability, and only a small amount of those contained in both fruits and vegetables is adsorbed by humans and become available in tissues. In order to avoid these drawbacks, their supplementation at the systemic level by nutraceuticals and at the skin level by cosmeceuticals is needed to ensure adequate amounts of these substances in the different tissues [17,18]. Cosmeceuticals containing natural antioxidants could ensure the adequate protection of the skin from oxidative stress, especially if properly formulated. Indeed, formulations have strategic importance in the development of effective products, especially when nanosystems are used, since they address considerable advantages, permitting to design a new generation of high-quality natural cosmetics, which maximize the effects of plant-derived components in the skin [19,20,21]. Among the different nanosystems, core–shell ones such as liposomes, lipid nanoparticles and nanoemulsions are particularly suitable for this purpose, since they have a surrounding stratum, which enhances the (i) stability against stressing conditions, (ii) skin penetration ability and (iii) antioxidant activity of loaded molecules [22,23,24,25]. Nanoemulsions, widely used for the delivery of antioxidants, are stable colloidal nanosized dispersions of two immiscible liquids (e.g., water and oil) stabilized by the appropriate surfactants and cosurfactants, which can be either natural or synthetic [26,27]. Natural surfactants are preferred by modern consumers, and their use is mandatory to obtain a whole natural formulation, but despite their promising properties, they are not always enough to effectively stabilize the system.

In the present study, aiming at preparing a cosmetic antioxidant by using natural components, the antioxidant phytocomplexes obtained from grape pomace of Sardinian white (Nasco) or red (Bovale) grape cultivars were loaded, alone or in association, in nanoemulsions prepared with water, natural oils, soy lecithin and, alternatively, a natural (Olivem^®^1000) or a synthetic (Kolliphor^®^RH40) surfactant. The physicochemical properties (mean diameter, polydispersity index, surface charge and storage stability); technological features (pH, stability under stressing conditions and UV irradiation) and biological behavior (in vitro biocompatibility, cell defense against oxidative damages and inhibition of nitrite release in cells) were evaluated to select the most promising combination.

## 2. Materials and Methods

### 2.1. Materials

Cyanidin, peonidin, luteolin 7–glucoside, quercetin, quercetin dihydrate, quercetin glucopyranoside, coumaric acid, gallic acid, syringic acid, epicatechin, kaempferol, Folin–Ciocalteu, Kolliphor^®^RH40 (kolliphor), methanol, ethanol, 3-(4,5-dimethylthiazol-2-yl)-2,5-diphenyltetrazolium bromide (MTT) tetrazolium salt and all other products of analytical grade were purchased from Sigma-Aldrich (Milan, Italy). Soy lecithin was purchased from Galeno Srl (Carmignano, Italy). Curù^®^, a natural oil, was kindly provided by Curù^®^Skin (Toronto, ON, Canada). Olivem^®^1000 (olivem) was purchased from Hallstar Italia (Arcore, Italy). Fetal bovine serum, penicillin, streptomycin, medium and the other reagents and plastics, unless otherwise specified, used for the in vitro cell culture studies were supplied by Thermo Fisher Scientific Inc. (Waltham, MA, USA). Water was purified through a Milli-Q system from Millipore (conductivity: 18.2 MΩ cm at 25 °C; Milford, MA, USA).

### 2.2. Production of Grape Pomace Extracts

Nasco and Bovale pomace, kindly provided by a local winery (Argiolas SpA, Serdiana, Cagliari, Italy) right after the winemaking process (2021), was stocked in a ventilated air dryer at 40 °C for 48 h and grinded to obtain a powder with small particles and a higher specific area. Anhydrous pomace (10 g) was dispersed in 200 mL of a mixture of ethanol and water (50:50 *v*/*v*), left for 24 h at 40 °C and finally centrifuged (4000× *g* rpm for 15 min) to separate the extractive solution from the exhausted biomass. Ethanol was removed from the solution through low-pressure evaporation using a rotary evaporator (Rotavapor RII, BÜCHI Labortechnik AG, Flawil, Switzerland), and the water was removed by freeze-drying to obtain a solid extract, which was stored in the dark in vacuum-packed glass containers until use.

### 2.3. Characterization of the Extracts

#### 2.3.1. Folin–Ciocalteu and DPPH Colorimetric Assays

The determination of the total phenolic content of the extracts obtained from Nasco and Bovale pomace was performed by the Folin–Ciocalteu assay. Each dry extract was dissolved in ethanol (1 mg/mL), and the solution (100 μL) was mixed with a solution containing the reagent (500 μL) and sodium carbonate solution (20% *w*/*v*, 1 mL). Milli-Q water was added up to 10 mL in a glass flask, and then, the mixture was vigorously stirred for 1 min and, finally, incubated in the dark at room temperature for 90 min. Samples were analyzed using a UV spectrophotometer at a wavelength of 750 nm. A 5-point calibration curve was generated using different concentrations of gallic acid (from 2 to 0.01 mg/mL), and the results were expressed as milligram of gallic acid equivalents/kg of dry extract.

The antioxidant potential of the extracts was assessed measuring their ability to scavenge 1,1-diphenyl-1-picrylhydrazil (DPPH) radicals. Each dry extract was dissolved in ethanol (1 mg/mL), the solution diluted with ethanol (1:50), mixed (20 µL) with DPPH (1980 μL) and methanolic solution (40 µg/mL) and incubated in the dark at room temperature for 30 min. The absorbance of the resulting solution was measured at 517 nm against a blank. The antioxidant activity was calculated according to the following equation:

antioxidant activity (%) = [(ABS_DPPH_ − ABS_sample_)/ABS_DPPH_] × 100.

#### 2.3.2. Identification and Quantification of Phenolic Components by HPLC

The identification and quantification of the phenolic compounds was carried out using an Agilent HPLC 1100 associated with a diode array detector and a computerized data integration system (ChemStation, Agilent). The separation was performed at room temperature by using a Kinetex C18 column (5 μm, 150 mm × 4.6 mm) (Phenomenex, Casalecchio di Reno, BO, Italy). The mobile phase was composed of phosphoric acid 0.22 M (A), and acetonitrile/methanol (50/50, *v*/*v*) (B), according to the following gradient: T_1_ = 0 96% A and 4% B, T_2_ = 40 50% A and 50% B, T_3_ = 45 40% A and 60% B, T_4_ = 70 0% A and 100% B, T_5_ = 71 96% A and 4% B and T_1_ = 81 96% A and 4% B. The flow rate was set at 0.3 mL/min, whereas the injection volume was 20 µL. The flavan-3-ols, hydroxybenzoic acids, hydroxycinnamic acid derivates, flavonoids and anthocyanins were monitored at 220 nm, 280 nm, 313 nm, 360 nm and 520 nm, respectively. The molecules were identified as a function of the retention times in comparison with that of the commercial standards and the UV–Vis spectra. The calibration curves were obtained using the external standard method, correlating the area of the peaks with the concentration. The grape pomace extracts were diluted 1:2 with methanol, shook for 1 min in a vortex and centrifuged for 10 min at 4000× *g* rpm. The obtained supernatants were injected into HPLC without further purification. All data were reported as mg/kg DW (dry weight).

### 2.4. Preparation of Nanoemulsions

Nasco (7.5 mg/mL) or Bovale grape pomace (7.5 mg/mL) or both (7.5 mg/mL each), a commercial mixture of natural oils (Curu^®^ containing grape, almond, lemon and pomegranate oils, 100 mg/mL); kolliphor (100 mg/mL) or, alternatively, olivem (10 mg/mL) and soy lecithin (25 mg/mL) were weighed in a glass vial, heated at 50 ± 0.5 °C in a thermostatic water bath and maintained under stirring for 20 min to obtain a homogeneous phase. Water was heated at 50 ± 0.5 °C as well, and gently poured over the oil phase. The dispersions were immediately sonicated at room temperature and without any further temperature control (20 cycles, 5 s on and 2 s off, 6.5 µ of probe amplitude/mL of sample and a probe frequency max of 23 kHz) using an exponential probe and a high-intensity ultrasonic disintegrator (Soniprep 150, MSE Crowley, London, UK). These conditions were the most suitable to avoid overheating of the sample and obtaining a small droplet size and stable transparent or milky nanoemulsions that were stored at room temperature (25 ± 1 °C) and protected from light until further characterization.

### 2.5. Characterization of Nanoemulsions

The average diameter and polydispersity index of the droplets were measured by photon correlation spectroscopy using a Zetasizer Ultra (Malvern Instruments, Worcestershire, UK). The Zetasizer Ultra was also used to measure the surface charge (zeta potential) by means of the measure of the electrophoretic mobility in dispersion by using the mixed-mode measurement-phase analysis (M3-PALS). Each sample was diluted (1:100) to be optically clear and to avoid the attenuation of the laser beam by the particles, along with the reduction of scattered light that can be detected [28]. The antioxidant activity of the nanoemulsions was measured by the DPPH colorimetric assay: 20 µL of each sample were dissolved in 1980 µL of DPPH methanolic solution (40 µg/mL) and incubated at room temperature for 30 min in the dark. Then, the absorbance was measured at 517 nm against the blank. The total phenolic content of the formulations was measured with Folin–Ciocalteu, according to the method reported in Section 2.3.1. The average size, the polydispersity index, the surface charge and the pH of the nanoemulsions were monitored for a period of 90 days of storage at room temperature to evaluate the stability of the samples over time.

### 2.6. Stability of Nanoemulsions under Stress Conditions

The nanoemulsions were stored at 25 °C for 30 days and, at 0 and 30 days, were subjected to stressful conditions: 1 h at 4 °C, 1 h at 50 °C and 30 min of centrifugation at 2500× *g* rpm. At the end of the treatment, the mean diameter, polydispersity index, surface charge, pH and antioxidant activity were measured.

### 2.7. UV Stability Testing

The stability of the extracts in dispersion or loaded into the nanoemulsions treated with a laminar flow hood of UV light was evaluated as well. Each sample (1 mL) was poured into a multiplate and exposed to UV light for 4 h. An aliquot of dispersions or nanoemulsions was taken at regular intervals (15 min, 30 min, 1 h, 2 h and 4 h), and the total phenolic content was measured. The percentage of degradation was calculated as a function of the variation of the phenolic content in comparison with untreated samples (100%).

### 2.8. In Vitro Cytotoxicity of Formulations

Some 75 cm^2^ flasks were used to let keratinocytes (HaCaT) grow as monolayers using an incubator set at 37 °C, 100% humidity and 5% carbon dioxide and Dulbecco’s Modified Eagle’s Medium with high glucose and L-glutamine, supplemented with 10% of fetal bovine serum and 1% of penicillin and streptomycin (10,000 units/mL of penicillin and 10,000 µg/mL of streptomycin) as the growth medium. Cells were seeded into 96-well plates at a density of 7.5 × 10^3^ cells/well, incubated for 24 h and then treated with the extracts in aqueous dispersion or loaded in nanoemulsions properly diluted with the cell medium to achieve the desired extract concentrations (7.5, 0.75 and 0.075 μg/mL). After 48 h of incubation, the medium was replaced with MTT (3(4,5-dimethylthiazolyl-2)-2, 5-diphenyltetrazolium bromide) (100 μL, 0.5 mg/mL final concentration). Three hours later, the MTT solution was removed, the formed formazan crystals were dissolved with dimethyl sulfoxide and the absorbance was measured at 570 nm with a microplate reader (Synergy 4 Reader, BioTek Instruments, AHSI S.p.A, Bernareggio, Italy). To verify the noninterference of the extracts, the same experiment was also performed by seeding the extract in dispersion in cell-free plates [29]. The experiments were repeated at least three times, each time in triplicate. The results are reported as cell viability (%) in comparison with untreated control cells (100% viability).

### 2.9. In Vitro Protective Effect of Formulations against Oxidative Damage in Keratinocytes

The effective protection of keratinocytes, by the extract, against damages induced using hydrogen peroxide was evaluated. Ninety-six-well plates were used to seed the cells (5 × 10^3^ cells/well), which were incubated at 37 °C in 5% carbon dioxide for 24 h. The cells were stressed with hydrogen peroxide (30% diluted 1:30,000 *v/v* with the medium) and immediately treated for 4 h with the extract in dispersion or loaded in nanoemulsions opportunely diluted to reach 7.5 and 0.75 µg/mL of each extract. Cells stressed with hydrogen peroxide and untreated were used as the negative control, while healthy, unstressed and untreated cells were used as the positive control. At the end of the experiment, the cells were washed with phosphate buffer solution, and the MTT assay was used to assess the viability, as reported above.

### 2.10. Inhibitory Effect of Formulations in Nitric Oxide Generation by Macrophages

RAW-264.7 were preincubated for 1 h with each formulation (extract in dispersion or loaded in nanoemulsions) and opportunely diluted to reach 7.5 and 0.75 µg/mL of each extract. The lipopolysaccharide (1 μg/mL final concentration) was added to each well and incubated for 20 h at 37 °C and 5% carbon dioxide. After incubation, the cell culture medium (100 μL) was withdrawn and transferred into a new 96-well plate and mixed with Griess reagent solution (100 μL), and the absorbance was measured at 540 nm using a microplate reader to calculate the nitrite content [28]. The inhibition of nitrite release was estimated as the ratio between the nitrite released by cells damaged and treated with formulations versus that released by damaged and untreated cells, considered 0% of nitrite inhibition.

### 2.11. Statistical Analysis of the Data

The results are expressed as the mean ± standard deviation. Analysis of variance (ANOVA) was used for multiple comparisons of the means, and Tukey’s test and Student’s *t*-test were performed to substantiate differences between groups using XL Statistics for Windows. The differences were considered statistically significant at *p* < 0.05.

## 3. Results

### 3.1. Characterization of Extracts

**Figure 1:**
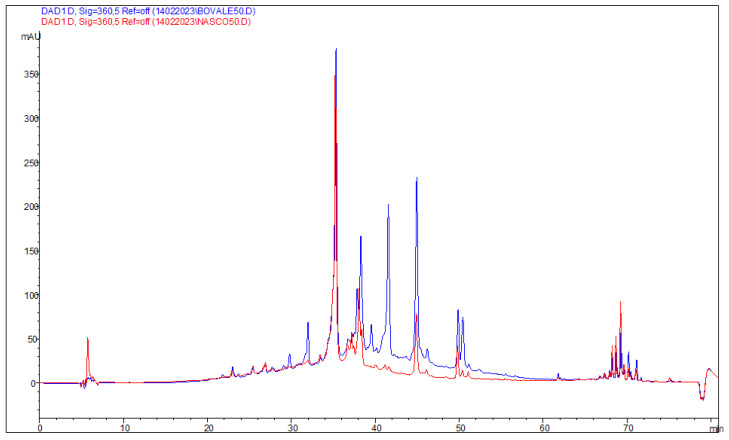
Chromatogram of the Bovale and Nasco extracts recorded at 360 nm.

The obtained extraction yield from Bovale pomace was ≈8% and that from Nasco pomace was lower, ≈5.5%. The obtained extracts had a comparable phenolic profile involving 12 common molecules having the same retention times and UV–Vis spectra (Table 1). The number of total polyphenols detected in Bovale extract was ≈ 46,280 mg/kg, about three-fold higher than that detected in the Nasco extract (≈15,842 mg/kg) (Table 1 and Figure 1). The Nasco extract contained 15 different polyphenols, 5 anthocyanins and 5 flavonoids and 1 hydroxycinnamic acid at low concentrations, while two flavan-3-ols and two hydroxybenzoic acids were contained at higher concentrations (≈1717 mg/kg and ≈10,307 mg/kg) in comparison with the Bovale extract (≈1291 mg/kg and ≈9427 mg/kg). The most concentrated polyphenols detected in Nasco extract were two flavan-3-ol compounds (6005 mg/kg and 4302 mg/kg), which were not identified, followed by quercetin glucopyranoside (1977 mg/kg). The Bovale extract contained 14 total polyphenols and 1 hydroxybenzoic acid detected in a lower amount in comparison with the Nasco extract, whereas the anthocyanins (five), the flavonoids (five) and the unique hydroxycinnamic acid resulted in higher concentrations.

The concentration of anthocyanins in the Bovale extract (≈30,355 mg/kg) was 46 times higher than that measured in the Nasco extract (≈654 mg/kg). In addition, an unknown anthocyanin derivative with a retention time of 41.54 was the most concentrated polyphenol (≈24,174 mg/kg), consisting of 50% of the total phenolic content. Other polyphenols contained in high amounts were epicatechin (≈5289 mg/kg) and quercetin glucopyranoside (≈2075 mg/kg). The total phenolic content was ≈10,012 ± 13 mg of gallic acid equivalent/kg of dry Nasco extract and ≈41,700 ± 10 mg gallic acid equivalent/kg of dry Bovale extract measured using the Folin–Ciocalteu colorimetric method. The antioxidant activity of the Nasco extract assessed with the DPPH colorimetric assay was ≈67% and that of the Bovale extract was ≈88%.

### 3.2. Characterization of Nanoemulsions

**Figure 2:**
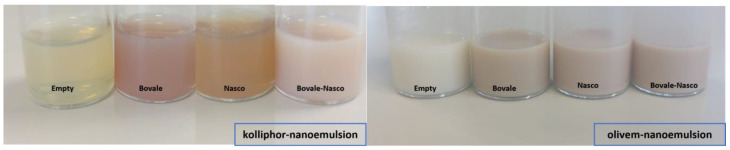
Representative images of kolliphor (**left panel**) and olivem nanoemulsions (**right panel**), empty or the loaded Bovale extract, Nasco extract or their combination.

The two extracts alone or in association were solubilized in a commercial mixture of natural oils and nanodispersed in water using soy lecithin as the cosurfactants and Olivem^®^1000 or, alternatively, Kolliphor^®^RH40 as the main surfactant. The first was selected due to its natural origin to obtain a completely natural formulation, while the latest was chosen because of its semisynthetic origin and optimal emulsifying properties, which, actually, led to the formation of a transparent dispersion, which became pink transparent when one extract was loaded and milky when both extracts were coloaded (Figure 2). The olivem nanoemulsions, either empty or loading the extracts, appeared milky during the visual inspection, indicating the formation of larger droplets (Figure 2).

The droplets of empty kolliphor nanoemulsion sized ≈65 nm and those of empty olivem nanoemulsions were slightly larger, ≈71 nm. Despite their different macroscopic aspect, the two nanoemulsions were monodispersed (polydispersity index 0.19) and negatively charged (≈−43 mV) (Table 2). The addition of the extracts in kolliphor nanoemulsions, irrespective of the used extracts or their combination, led to a slight increase in the droplet size up to ≈81 nm (*p* > 0.05 among the droplet sizes of kolliphor nanoemulsions loading the two extracts alone or their association; *p* < 0.05 versus the droplet size of empty kolliphor nanoemulsion) and the polydispersity index up to 0.26. The assembling of olivem nanoemulsions was affected to a greater extent by the addition of the extracts, particularly by their combination; indeed, the droplets of Nasco olivem nanoemulsion and Bovale olivem nanoemulsion were sized ≈119 nm, while those of Nasco–Bovale olivem nanoemulsions were ≈148 nm (*p* < 0.05 versus the droplet size of an empty olivem nanoemulsion; *p* > 0.05 versus the droplet size of Nasco olivem nanoemulsions and Bovale olivem nanoemulsions). The polydispersity index of olivem nanoemulsions loading only Nasco or only Bovale was low, ~0.13, and that of olivem nanoemulsions loading the two extracts was higher (~0.29). The droplets were always negatively charged, irrespective of the used extract.

The antioxidant activity of the formulations was mostly affected by the loaded extracts and, to a lesser extent, by the surfactant used; indeed, it was ~70% when the Bovale extract was loaded and increased up to ~81% when both extracts were coloaded, while it was lower when the Nasco extract was loaded, being ~53% that of kolliphor nanoemulsions and ~62% that of olivem nanoemulsions. Similarly, the pH was mostly affected by the used extract, rather than the surfactant used, as it was ~6.0 for empty nanoemulsions and decreased to ~5.5 for the loading Nasco extract, to ~5.1 loading for the Bovale extract and reached the lower value of ~4.8 when co-loading the two extracts, indicating the acidic pH of the extracts due to the presence of organic and inorganic acid, which make them suitable for topical application.

**Figure 3:**
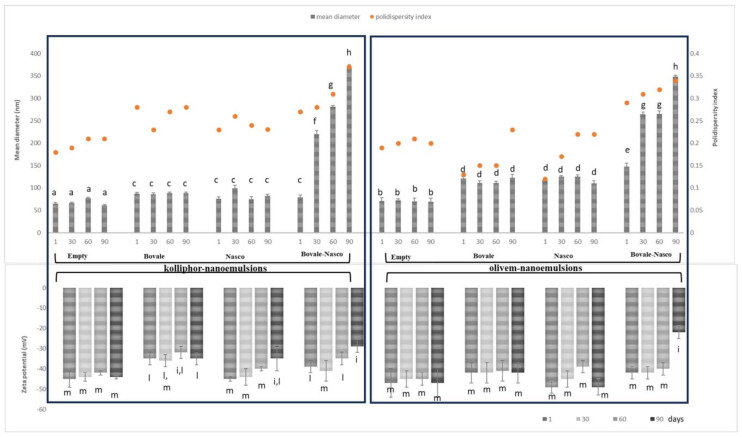
Mean diameter (nm), polydispersity index and zeta potential (mV) of kolliphor and olivem nanoemulsions loading Bovale, Nasco or their combination stored for 90 days at 25 °C. Data are reported as the mean values ± standard deviations (error bars) (n = 3). The same letter (a–i, l, m) indicates that the mean diameter values (upper panel) and zeta potential values (lower panel) are not statistically different from each other (*p* > 0.05) and different from other values (*p* < 0.05).

To evaluate the stability of the formulations, the mean diameter, polydispersity index, zeta potential, antioxidant activity and pH were monitored for 90 days (Figure 3). The behavior of kolliphor or olivem nanoemulsions was similar, and the nanoemulsions loading Bovale and Nasco were almost stable, as the tested parameters remained constant or underwent small variations; on the contrary, the nanoemulsions co-loading Bovale and Nasco were unstable as the parameters progressively increased: the mean diameter up to ≈370 nm, the polydispersity index up to ≈0.37 and the zeta potential up to ≈−25 mV, suggesting that the association of the two extracts caused a variation of the component’s assembling, thus significantly reducing the stability of the system. The antioxidant activity and pH of the formulations did not change during the storage (data not shown), probably because these parameters were not dependent on the nanoemulsion structure but were mainly connected with the extract contained.

### 3.3. Stability of Nanoemulsions under Stress Conditions

**Figure 4:**
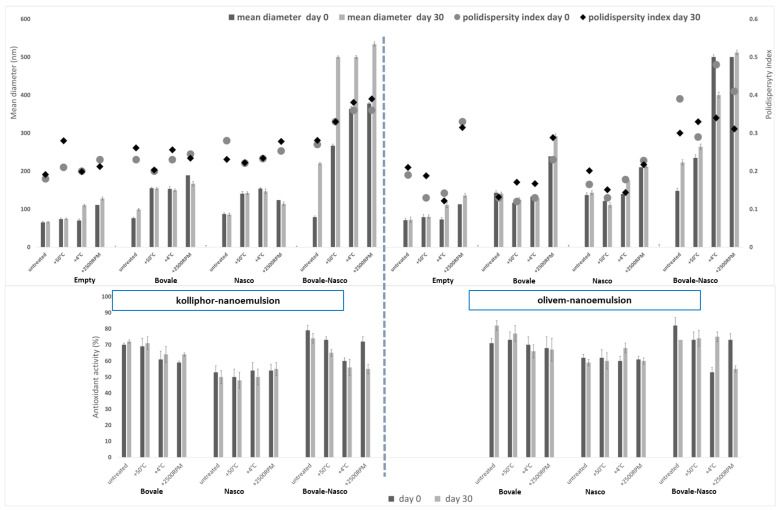
Upper panel: mean diameter (bars) and polydispersity index (dots and rhombuses) of kolliphor and olivem nanoemulsions loading Bovale, Nasco or their combination stored at 25 °C for 30 days and treated (at times 0 and 30) 1 h at 50 °C, 1 h at 4 °C and 30 min at 2500× *g* rpm (centrifugation). Lower panel: antioxidant activity of kolliphor and olivem nanoemulsions loading Bovale, Nasco or their combination stored at 25 °C for 30 days and treated (at times 0 and 30) 1 h at 50 °C, 1 h at 4 °C and 30 min at 2500× *g* rpm (centrifugation). Mean values ± standard deviations (error bars) are reported (n = 3).

Phase separation, like sedimentation, flotation or creaming, usually happens during liquid dispersions driven by the Earth’s gravity and can be considerably accelerated by mechanical and thermal stimuli such as centrifugation or heating [30]. The application of these external stresses permits to evaluate the long-term stability of colloidal liquid formulations as a function of their applications (Figure 4) [31]. In order to do this, nanoemulsions, freshly prepared and stored for 30 days, were subjected to thermal and mechanical destabilizing conditions (50 °C for 1 h, 4 °C for another h and then centrifugation for 30 min at 2500× *g* rpm). The droplet size of the empty nanoemulsions (irrespective of the used surfactant) was only affected by the thermal stress at 4 °C, and the centrifugation was measured at 2500× *g* rpm, while the polydispersity index increased already after the incubation at 50 °C, suggesting a variation of the homogeneity of the system, probably due to the aggregation of the droplets. The droplet size of kolliphor nanoemulsions loading either Bovale or Nasco alone slightly increased up to ≈170 nm, while the polydispersity index remained almost constant; when Bovale and Nasco were coloaded, the droplet size strongly increased up to ≈500 nm, especially when the treatment was performed after 30 days of storage. Their polydispersity index increased as well, indicating the formation of a polydisperse system that can be associated with aggregation phenomena. The behavior of olivem nanoemulsions was similar, and the most significant changes in the tested parameters were observed when Bovale and Nasco were coloaded. According to these measurements, from a macroscopic point of view, no phase separation was observed at the end of the destabilizing process, except when Bovale and Nasco were coloaded in nanoemulsions, probably because the used amount reached a critical and higher concentration that could be loaded, confirming the low suitability of this association. The zeta potential and pH values remained unchanged after each step of the destabilizing process (data not shown), confirming that these parameters were mostly dependent on the acidic fraction of the extract, which was not affected by the treatment.

The antioxidant activity of Nasco-loaded nanoemulsions was the lowest, followed by that of nanoemulsions loading Bovale and their association. Moreover, the antioxidant activity of Nasco remained constant, while the other slightly decreased.

**Figure 5:**
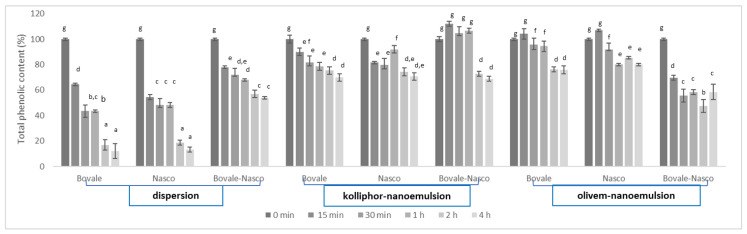
The total phenolic content of kolliphor and olivem nanoemulsions loading Bovale, Nasco or their combination treated with ultraviolet radiation for 4 h. Data reported as the mean values ± standard deviations (error bars) (n = 3). The same letter (a–g) indicates not statistically different values (*p* > 0.05), which are instead statistically different from values marked with different letters (*p* < 0.05).

The UV stability tests were performed to estimate the effects of exposure to sunlight on the extract properties, in terms of the total phenolic content, and the protective role played by the nanoemulsions. To evaluate the rate of degradation of the extracts, the total phenolic content was measured, at scheduled time points by means of the Folin–Ciocalteu colorimetric test, using the extracts in dispersion as comparison (Figure 5). The phenolic content of the extracts in dispersions strongly decreased as a function of the time and, at 4 h of irradiation, was ~20% of the initial amount, suggesting a significant degradation of the phenolic molecules contained in the extract. When the extracts were loaded in nanoemulsions, either alone or in association, the decrease was less intense, and, at 4 h, the phenolic content was ~80%; irrespective of the used extract or surfactant, only that of Bovale–Nasco coloaded in olivem nanoemulsion was slight lower, ~60%, probably due to the higher instability of these systems in comparison with those containing only one extract. The results underline the ability of both nanoemulsions to slow down the extract degradation caused by the exposure to UV radiations.

### 3.4. In Vitro Cytotoxicity of Nanoemulsions and Protection of Cells against Oxidative Damage

**Figure 6:**
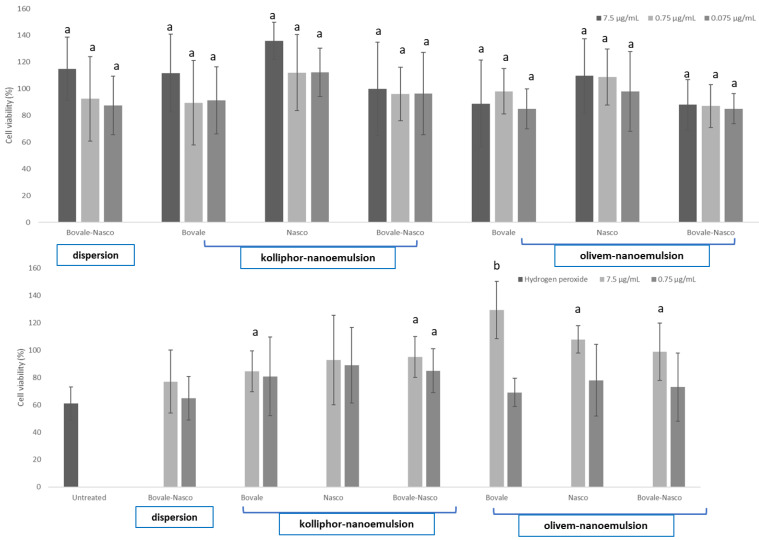
Viability of keratinocytes (upper panel) incubated for 48 h with Nasco and Bovale in dispersion or loaded in kolliphor and olivem nanoemulsions properly diluted with growth medium up to 7.5, 0.75 and 0.075 μg/mL. Viability of keratinocytes (lower panel) stressed with hydrogen peroxide and immediately treated with Nasco and Bovale in dispersion or loaded in kolliphor and olivem nanoemulsions properly diluted with growth medium up to 7.5 and 0.75 μg/mL. The mean values (bars) ± standard deviations are reported (n = 3). The same letter indicates a value not statistically different from each other and different from the viability of cells stressed with hydrogen peroxide and untreated (*p* > 0.05).

The viability of the cells incubated with extracts in dispersions was 83% and not statistically different from that of the cells incubated with extracts loaded in nanoemulsions, confirming their biocompatibility (Figure 6, upper panel). Stressing the cells with hydrogen peroxide, a significant decrease in cell viability, up to ~62%, was detected (Figure 6, lower panel). Their simultaneous protection with Bovale and Nasco in dispersion led to a slight increase in the cell viability up to ~78%, but the values were not statistically different (*p* > 0.05 versus the value of cells stressed with hydrogen peroxide and untreated). Using 7.5 μg/mL of Bovale or Bovale–Nasco loaded in kolliphor and olivem nanoemulsions and Nasco olivem nanoemulsion, the viability was ≈98% and statistically different from that of the cells stressed with hydrogen peroxide and untreated (*p* < 0.05), suggesting that the association of the two extracts was not correspondent to an increased beneficial effect. Olivem nanoemulsions loading the Bovale extract were ensured the highest protection, as the viability was ~120% and statistically different from the other values (*p* < 0.05), indicating the optimal ability of this formulation in protecting the cells. In this case, the beneficial properties of the Bovale extract were improved by its loading into a nanoemulsion, which may ensure a better interaction with cells and an improved exertion of its antioxidant activity to a better extent than the free extract, thus effectively counteracting the oxidative stress and promoting cell proliferation.

### 3.5. Inhibition of Nitric Oxide Generation in Macrophages

**Figure 7:**
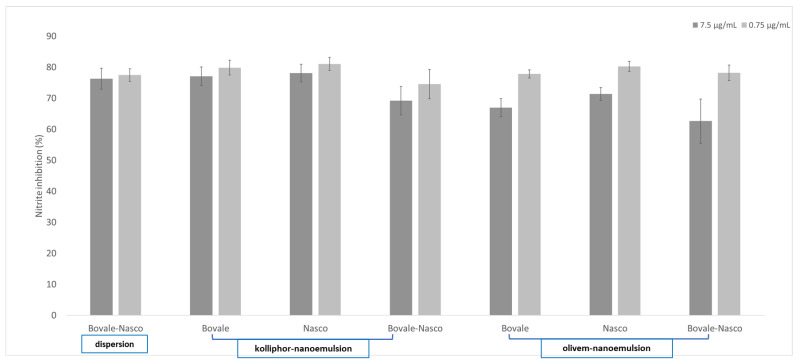
Nitrite release inhibition in cells damaged with lipopolysaccharide and pretreated with Nasco and Bovale in dispersion or loaded in kolliphor and olivem nanoemulsions and properly diluted with growth medium to 7.5 and 0.75 µg/mL. The mean values ± standard deviations (error bars) are reported (n = 6).

Macrophages damaged with lipopolysaccharide, which is considered the major component of the outermost membrane of Gram-negative bacteria, are characterized by an overexpression of nitric oxide that has been demonstrated to be associated with the pathogenesis of acute and chronic inflammatory conditions [32]. RAW-264.7 was preventively pretreated with formulations and then with lipopolysaccharide, and the nitric oxide production was quantified (Figure 7). The pretreatment with Nasco and Bovale extracts in dispersion inhibited the nitric oxide release up to ~80%, but in this case, any advantaged was provided by their loading in nanoemulsions as the release remained unchanged.

## 4. Discussion

Nanoemulsions are biphasic systems highly appreciated by both cosmetic industries and final consumers; however, they need to be stabilized by specific additives, such as surfactants, that can be chosen between synthetic and/or natural. Given that, two different formulations were prepared and compared, using Kolliphor^®^RH40 or Olivem^®^1000 as representatives of synthetic and natural surfactants, to obtain a final product characterized by high stability, appropriated textural properties and performances.

In the present study, using a whole green approach, valuable antioxidant extracts were obtained from the pomace of Nasco and Bovale, valorizing agri-food residues, transforming them into new resources and approaching towards the zero waste goal [33,34,35,36]. The extract obtained from pomace of the autochthonous red variety had a higher phenolic content (three times more) than that from the white variety (Nasco), probably due to the anthocyanins, largely contained in red grapes and responsible for their color, as already reported [23,37,38]. Each extract was loaded in nanoemulsions at a high concentration (7.5 mg/mL), and they were also combined (coloaded) in the same formulation (7.5 mg/mL each) to maximize the beneficial effect. The obtained results underlined that the combination of the two extracts was not helpful, probably because the high final concentration (15 mg/mL) was difficult to be dispersed in the systems and caused instability, especially under stressing conditions; moreover, the combination of the two extracts did not address any advantage if compared to the formulations loading the single extract. Formulations were tailored for skin delivery by using eudermic and, as far as possible, natural components such as water, a commercial mixture of natural oils (Curu^®^), soy lecithin and, alternatively, two different surfactants: Olivem^®^1000 and Kolliphor^®^RH40. Two different surfactants were tested because their role is crucial in sealing the fate of the nanoemulsions and may strongly affect their stability and skin delivery performances [39]. The first permits to obtain a whole natural formulation since its origin and classification, and the second, derived from natural castor oil modified by pegylation, is well known as an effective emulsifier in the pharmaceutic and cosmetic fields [40,41,42]. Both surfactants permitted to formulate an optimal anti-age, antioxidant and protective cosmetic formulation with a different appearance, as kolliphor nanoemulsions were transparent and especially suitable as a cosmetic serum, while olivem nanoemulsions were milky and ideal as cosmetic milk. From the microscopic point of view, both formulations were homogeneous, nano-sized, stable and able to protect the extract from the degradation caused by the exposure to UV radiation, prolonging the beneficial effect of the extract on the skin. In agreement with previous findings, there was not a direct correlation with the used surfactant and the protective effect of the systems [6,43].

The loading of the extracts slightly affected the mean diameter, which became larger, especially for olivem nanoemulsions, according to the results obtained by Zorzi et al. 2016, which found that the loaded extract increased the droplet sizes of nanoemulsions [44]. Anyway, the small differences found in the droplet size did not affect the system stability, only the loading of the two extracts, reaching the critical concentration to be loaded and causing destabilizing effects after the thermal stress and centrifugation, according with the results of Gadkari et al. 2017, which found that thermal stress increase the droplet size and homogeneity of nanoemulsions [45]. At the same time, combining the extracts in the same formulation did not produce the expected synergistic benefits, as the UV protection and antioxidant defense of the extract did not increase. The pH of the formulations was subtly affected by the extracts and their content, as Bovale nanoemulsions had a slightly lower pH value (~5.1) than Nasco nanoemulsions (~5.4) due to the organic and inorganic acidic molecules contained in the extract, and both seem to be suitable for skin application [7]. All tested nanoemulsions were highly biocompatible against HaCaT cells, and only the highest concentrations used (7.5 μg/mL in the cell well) were capable of protecting the cells from the damages and death caused by hydrogen peroxide, reestablishing the healthy condition. In particular, Bovale olivem nanoemulsions address an optimal protection and also improve the cell proliferation, disclosing a regenerative effect on the skin. All nanoemulsions inhibited (~80%) nitrite release, which provided additional protection against inflammatory processes.

## 5. Conclusions

Overall, the results indicate that the extracts obtained from Bovale and Nasco pomace are optimal antioxidant and antiaging materials to be individually loaded in nanoemulsions, while their co-loading did not address any advantage. Using Olivem^®^1000 is possible to obtain a total natural formulation, having an ideal pH (~5.1) for skin application; the ability to protect the extract from UV degradation and, especially, loading Bovale in olivem nanoemulsions is possible to improve the cell protection from oxidative stress. The last formulation seems to be ideal to prepare a pinkish cosmetic milk, highly appreciated by consumers not only for its pleasant color but, especially, for its effective skin protection. These preliminary results have significant implications, especially considering the ecological importance of using natural and sustainable materials in industrial applications.

## Figures and Tables

**Table 1:** Phenolic profile (grouped on anthocyanins, flavonoids, hydroxycinnamic acids, hydroxybenzoic acids and flavan-3-ols) of the Bovale and Nasco extracts (mg/kg of dry extract): ^a^ quantified as cyanidin; ^b^ quantified as luteolin 7—glucoside; ^c^ quantified as quercetin; ^d^ quantified as coumaric acid; ^e^ quantified as gallic acid. The mean values ± relative standard deviation (RSD) were reported.

Bovale			Nasco		
**Anthocyanins**	TR (min)	mg/kg ± RSD%	**Anthocyanins**	TR (min)	mg/kg ± RSD%
Peonidin 3 glucopyranoside	29.07	2010.6 ± 1.3	Peonidin 3 glucopyranoside	29.07	<LOQ
Malvidin 3 glucoside ^a^	29.8	1919.1 ± 3.2	Malvidin 3 glucoside ^a^	29.79	39.1 ± 1.1
Anthocyanins derivative ^a^	37.28	625.9 ± 4.5	Anthocyanins derivative ^a^	37.24	<LOQ
Anthocyanins derivative ^a^	39.56	1625.5 ± 1.0	Anthocyanins derivative ^a^	39.52	29.9 ± 2.5
Anthocyanins derivative ^a^	41.54	24,174.4 ± 5.5	Anthocyanins derivative ^a^	41.62	585.8 ± 6.6
**Flavonoids**	TR (min)	mg/kg ± RSD%	**Flavonoids**	TR (min)	mg/kg ± RSD%
Quercetin glucopyranoside	35.32	2075.1 ± 3.4	Quercetin glucopyranoside	35.26	1977.7 ± 8.1
Luteolin derivative ^b^	38.28	1409.6 ± 8.5	Luteolin derivative ^b^	38.37	658.0 ± 8.0
Quercetin dihydrate	44.94	1162.8 ± 1.1	Quercetin dihydrate	44.89	412.0 ± 6.7
Kaempferol	49.87	103.5 ± 2.4	Kaempferol	49.8	61.4 ± 1.9
Quercetin derivative ^c^	50.44	181.7 ± 4.1	Quercetin derivative ^c^	50.39	19.8 ± 2.6
**Hydroxycinnamic acids**	TR (min)	mg/kg ± RSD%	**Hydroxycinnamic acids**	TR (min)	mg/kg ± RSD%
Cumaric acid derivative ^d^	25.41	273.0 ± 7.6	Cumaric acid derivative ^d^	25.38	34.2 ± 5.7
**Hydroxybenzoic acids**	TR (min)	mg/kg ± RSD%	**Hydroxybenzoic acids**	TR (min)	mg/kg ± RSD%
Gallic acid	13.08	1291.9 ± 3.3	Gallic acid	12.99	1462.2 ± 5.8
			Syringic acid	24.93	255.4 ± 4.2
**Flavan-3-ols**	TR (min)	mg/kg ± RSD%	**Flavan-3-ols**	TR (min)	mg/kg ± RSD%
Epicatechin	23.06	5289.1 ± 4.3	Unknown ^e^	23.19	6004.9 ± 7.1
Unknown ^e^	26.81	4138.6 ± 5.6	Unknown ^e^	26.66	4302.4 ± 3.4
**Total polyphenols**		**46,280.8 ± 4.8**	**Total polyphenols**		**15,842.8 ± 6.1**

**Table 2:** Mean diameter (MD), polydispersity index (PDI), zeta potential (ZP), antioxidant activity (AA) and pH of kolliphor and olivem nanoemulsions loading Bovale, Nasco or their combination. Each value represents the mean ± standard deviation of at least three replicates. The same letter (a–i) indicates values not statistically different from each other (*p* > 0.05) and different from other values (*p* < 0.05).

	MD (nm)	PDI	ZP (mV)	AA (%)	pH
Empty kolliphor nanoemulsions	65 ± 2	0.18	−45 ^e^ ± 1	-	6.1
Bovale kolliphor nanoemulsions	87 ^a^ ± 4	0.18	−35 ^d^ ± 4	70 ^h^ ± 2	5.1
Nasco kolliphor nanoemulsions	76 ^a^ ± 3	0.21	−45 ^d^ ± 1	53 ± 4	5.4
Bovale–Nasco kolliphor nanoemulsions	79 ^a^ ± 7	0.27	−39 ^e^ ± 7	79 ^i^ ± 3	4.8
Empty olivem nanoemulsions	71 ± 3	0.19	−42 ^f^ ± 3	-	6.0
Bovale olivem nanoemulsions	121 ^b^ ± 4	0.13	−47 ^g^ ± 3	71 ^h^ ± 3	5.0
Nasco olivem nanoemulsions	117 ^b^ ± 4	0.12	−49 ^g^ ± 3	62 ± 5	5.7
Bovale–Nasco olivem nanoemulsions	148 ^c^ ± 7	0.29	−42 ^f^ ± 1	82 ^i^ ± 2	4.9

## Data Availability

Not applicable.
